# Bayesian model to detect phenotype-specific genes for copy number data

**DOI:** 10.1186/1471-2105-13-130

**Published:** 2012-06-13

**Authors:** Juan R González, Carlos Abellán, Juan J Abellán

**Affiliations:** 1 Center for Research in Environmental Epidemiology (CREAL), Barcelona, Spain; 2 Institut Municipal d’Investigació Mèdica (IMIM), Barcelona, Spain; 3 Joint Research Unit on Genomics and Health, Centre for Public Health Research (CSISP) and Cavanilles Institute for Biodiversity and Evolutionary Biology, University of Valencia, Valencia, Spain; 4 CIBER Epidemiología y Salud Pública (CIBERESP), Spain

## Abstract

**Background:**

An important question in genetic studies is to determine those genetic variants, in particular CNVs, that are specific to different groups of individuals. This could help in elucidating differences in disease predisposition and response to pharmaceutical treatments. We propose a Bayesian model designed to analyze thousands of copy number variants (CNVs) where only few of them are expected to be associated with a specific phenotype.

**Results:**

The model is illustrated by analyzing three major human groups belonging to HapMap data. We also show how the model can be used to determine specific CNVs related to response to treatment in patients diagnosed with ovarian cancer. The model is also extended to address the problem of how to adjust for confounding covariates (e.g., population stratification). Through a simulation study, we show that the proposed model outperforms other approaches that are typically used to analyze this data when analyzing common copy-number polymorphisms (CNPs) or complex CNVs. We have developed an **R** package, called **bayesGen**, that implements the model and estimating algorithms.

**Conclusions:**

Our proposed model is useful to discover specific genetic variants when different subgroups of individuals are analyzed. The model can address studies with or without control group. By integrating all data in a unique model we can obtain a list of genes that are associated with a given phenotype as well as a different list of genes that are shared among the different subtypes of cases.

## Background

The aim of genome-wide association studies (GWAS) is to assess the association between single nucleotide polymorphisms (SNPs) and common diseases. Recent GWAS have been successful in discovering SNPs significantly associated with complex diseases [1,2]. However, published SNP associations account for only a fraction of the genetic component of most common diseases [3]. Lately, several studies have been focused on the association between copy number variants (CNV) and disease. Some reports have suggested a role of rare CNVs (i.e. CNV with low prevalence in the general population) in susceptibility to neurodevelopmental disorders [4-6]. Other studies have shown statistically significant associations between common CNVs (i.e. CNV with high prevalence in the general population) and common diseases such as psoriasis [7], Crohn’s disease [8], HIV-1/AIDS [9], or Alzeheimer’s disease [10] to name a few. These studies indicate that the identification of DNA copy number is important in understanding the genesis and progression of human diseases.

Several techniques and platforms have been developed for GWAS involving CNVs, such as array-based comparative genomic hybridization (aCGH). For targeted studies, other techniques such as real time PCR, or Multiplex Ligation-dependent Probe Amplification (MLPA) assays have been used to compare the copy number status of particular loci in cases and controls. In both cases, a signal intensity is measured for each CNV as a continuous variable, from which the copy number status is inferred. In many cases, the distribution of the observed CNV probe measurements is continuous and multimodal, representing the unobserved copy number status as a latent variable [11]. Thus, scoring copy number may lead to misclassification and, hence, unreliable results, making it necessary to incorporate uncertainty in the association analysis. So far, two methods have been developed to analyze CNV data that incorporate uncertainty. The first one performs the calling procedure and incorporates the posterior probabilities in a latent class model [11], while the other is based on a likelihood test that combines calling and testing in a single procedure [12].

Despite the existence of these methods, CNV association studies often analyze CNVs with very low uncertainty that are not likely genotyping artefacts. For example, in the GWAS performed in the Myocardial Infarction Genetics Consortium [13] the authors pointed out that: “[for the CNV analysis] as an initial quality control step, [they] removed any variant where more than 10% of the copy calls were uncertain” [13]. Another example is given in [14] were only CNVs without uncertainty are analyzed. Such approach allows the use of standard tests such as *χ*^2^, Fisher or Mann-Whitney tests [7-10] to assess differences between cases and controls.

In this article, we present a Bayesian shared component model for CNV-based association studies. We illustrate the model with a case study to determine those CNVs that are specific to a given population when comparing individuals belonging to the HapMap project. In this example it is expected to find differences in a large proportion of CNVs due to ethnic background. An example including patients with ovarian cancer is analyzed in order to illustrate how our model identifies phenotype-associated CNVs when a tiny number of CNVs are expected to be differente accross groups. Our approach adapts and extends the model suggested by [15] for genetic association studies based on SNPs to cope with CNVs too. We introduce the Bayesian shared component model formulation, the likelihood, priors and hyperpriors as well as the inferential process. We empirically examine its performance by using simulated data. We generated data under two scenarios in order to mimic the type of CNVs that are typically analyzed. The first simulation generates CNVs which can be tagged by SNPs (also known as copy number polymorphisms, CNPs), while the second one mimics situations in which complex CNVs are studied. The analyzed data sets and proposed methods are available in the R package bayesGenhttp://www.creal.cat/jrgonzalez/software.htm.

## Methods

### Data sets

The first motivating data were collected from a genetic study conducted at the Center for Genomic Regulation (CRG) in Barcelona, Spain. The study aimed to determine those CNVs that are specific to major human ethnic groups included in the HapMap project (e.g., African, Asian or European) [16] (http://hapmap.ncbi.nlm.nih.gov/). This type of data can help in the understanding of some Mendelian diseases such as cystic fibrosis [17] or deafness [18], that present different prevalences in the different populations. In addition, the genomic variants that are population-specific can guide to drug discovery. For example, the existing population variability in the acetylating activity of the N-acetyltransferase 2 (NAT2) gene makes possible to determine those ethnic groups that are more susceptible to develop some diseases [19].

The second motivating data belongs to an study on ovarian cancer. The data are obtained from The Cancer Genome Atlas (TCGA) data portal http://cancergenome.nih.gov/ and it includes phenotype and CNV information for 572 females. We are interested in determining those CNVs that are specific to each type of response to treatment. In order to address this problem, we analyzed the variable named *‘primary_therapy_outcome_success’* that contains information about the response for the first therapy received. Our final data set contains information for 456 females, since 116 of them did not have information for this variable. This variable had 4 categories: ’Complete remission’, ’Partial remission’, ’Stable disease’ and ’Progressive disease’. Categories ’Stable disease’ and ’Progressive disease’ were collapsed into one categorie (’Null response’). The copy number data matrix contains the number of copies for each CNV annotated at the Database of Genomic Variants using the genome build GRCh37 (http://projects.tcag.ca/variation/downloads/variation.hg19.v10.nov.2010.txt).

As previously mentioned, a very simple approach to determine the CNVs that are specific to each subgroup of individuals is to compare the observed CNV frequencies between individuals from different groups [7,16]. One of the main limitations of this approach is that the number of copies may vary between 0 and 6 and therefore *χ*^2^, Fisher or Mann-Whitney tests can be underpowered. In addition, most of the analyzed CNVs have similar frequencies accross ethnic groups, and only a few, if any, show differences between them. Therefore, the use of a shared component model can be very useful in the context of CNVs.

### The Bayesian Model

Let {*X*_*ijp*_ ∈ *D*} be the number of copies of the *j*th CNV, for the *i*th individual of population *p*, where *D* denotes the set of indices for the observed data, *i *= 1,…,*n* (number of individuals), *j *= 1,…,*c* (number of CNVs) and *p *= 1,…,*P*(number of populations). We assume that all individuals in the same population group have the same chance of having a number of copies in a given CNV, then we observe *X*_*ijp*_ ∈ {0,1,2,3,4,…}. The motivation for this assumption relies on the fact that we are looking for associations between CNVs and populations. If a given CNV is linked to a specific population, it is expected that most of the individuals in that population have similar values for that CNV.

Now, let $Y_{\text{jp}}=\overset{n_{\text{jp}}}{\underset{i=1}{\sum }}\frac{X_{\text{ijp}}}{n_{\text{jp}}}$ be the average number of copies found in the *j*th CNV of the *p*th population, where *n*_*jd*_ denotes the number of individuals in population *p* with non-missing information for the *j*th CNV. Then, by the central limit theorem [20], and assuming independence among individuals we have

(1) $$Y_{\text{jp}}\dot{∼}N(\mu_{\text{jp}},\nu_{p}^{2}),$$

where *μ*_*jp*_ is the mean number of copies for CNV *j* in population *p* and $\nu_{p}^{2}$ is the variation of the average of CNV frequencies in population *p*.

We introduce the next shared component formulation with Gaussian likelihood to decompose the variability of *μ*_*jp*_

(2) $$\mu_{\text{jp}}=\alpha_{p}+\beta_{p}\cdot \theta_{j}+\lambda_{\text{jp}},$$

where *α*_*p*_ is a population-specific intercept, *θ*_*j*_ is the component shared by all populations, *β*_*p*_ denotes the loading of the common component into population *p* and *λ*_*jp*_ encodes the population-specific components. In order to make the model as flexible as possible we have considered that $\nu_{p}^{2}$ depends on the population group *p*. However, a simpler model can also be fitted by considering that *Y*_*jp*_ has the same variance for each population group, *ν*^2^. The likelihood of our proposed model is

$$\begin{array}{ll} l(\alpha_{p},\beta_{p},\theta_{j},\lambda_{\text{jp}},\nu_{p}) & \propto \underset{p}{\prod }\underset{j}{\prod }\nu_{p}^{-1}\text{exp}\left(\nu_{p}^{-2}{\left(Y_{\text{jp}}-\alpha_{p}-\beta_{p}\theta_{j}-\lambda_{\text{jp}}\right)}^{2}\right)\\ & =\underset{p}{\prod }\nu_{p}^{-J}\text{exp}\left(\nu_{p}^{-2}\underset{j}{\sum }{\left(Y_{\text{jp}}-\alpha_{p}-\beta_{p}\theta_{j}-\lambda_{\text{jp}}\right)}^{2}\right) \end{array}$$

**Figure 1:**
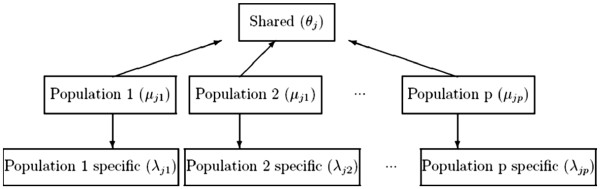
**Schematic representations of the shared component model using a symmetric formulation (**i.e.**, no reference group).** The index *j* denotes the *j*-th CNV and *p* is the number of groups.

Figure 1 depicts a schematic representation of our model. Notice that this formulation considers that no reference group is available (i.e control group). The formulation can be changed to accomodate the possibility of having a control group. For example, in the context of a case-control study where different diseases and only one group of control individuals is available. This is the case of the Wellcome Trust Case Control Consortium (WTCCC) study where 7 common diseases are compared with a unique group of controls [21] and thousands of CNVs were analyzed.

In the Bayesian framework, all parameters must be assigned prior distributions that, in turn, may depend on new parameters, which are referred to as hyperparameters. Prior distributions (hyperpriors) must also be assigned to these. To complete the Bayesian formulation, the prior and hyperprior distributions for the model parameters are needed. Our basic principle in specifying these distributions is to let the data likelihood dominate over the prior information. To achieve this, it is common to consider prior distributions with large variances that allow for a really wide range of potential values for the parameters thus being non-informative a priori. Following this we chose flat prior distributions. We also refer to previous similar studies that specify prior distributions in this way. We assumed the following priors

$$\begin{array}{lll} \alpha_{p}\;\; & ∼\;\;\text{Normal}(0,1000)\; & \;\\ \theta_{j}\;\; & ∼\;\;\text{Normal}(0,\sigma_{\theta }^{2})\; & \;\\ \lambda_{\text{jp}}\;\; & ∼\;\;t_{4}(0,\sigma_{p}^{2})\; & \;\\ \beta_{p} & ∼\;\;\text{Normal}(0,100)\; & \; \end{array}$$

and non-informative hyperpriors for the standard deviations of the random effects

$$\sigma_{\theta },\sigma_{p}∼\text{Normal}(0,100)\cdot 1\;\;I_{\left(0,+\infty \right)}$$

For the sake of identifiability we fixed $\sigma_{\theta }^{2}=1$. These priors and hyperpriors are commonly used for full Bayesian statistical inference when information about the model parameters is not available. However, in order to account for large values, the specific components, *λ*_*jp*_, were considered as zero-mean *t*-distributions with 4 degrees of freedom and unknown variances. The priors and hyperpriors for the asymmetric formulation (e.g. having a control group and different diseases) are mainly the same, except that we consider *β*_1_ = 1, where *β*_1_ corresponds to the reference population.

#### Inclusion of covariates

In almost all situations the disease is affected not only by genetic factors but also by environmental determinants. In these situations the association between the disease and CNVs has to be adjusted by some covariates that indicate whether an individual is exposed or not to those environmental variables. Our model can accomodate this information in the case of having categorical covariates (e.g., exposed vs non-exposed, males vs females, smokers vs non-smokers, ...) by aggregating the data in more categories. For instance, suppose we have a categorical covariate *Z* taking values in a set of *K* categories. Then, we will have *P *×* K* groups and we aggregate the CNV counts over all of them: $Y_{\text{jkp}}=\underset{i}{\sum }X_{\text{ijkp}}$. The adjustment for *Z* could be introduced in the model as follows:

$$\begin{array}{lll} Y_{\text{jkp}} & ∼\text{Normal}\left(\mu_{\text{jkp}},\sigma_{\text{kp}}\right)\; & \;\\ \mu_{\text{jkp}} & =\alpha_{p}+\gamma_{k}+\beta_{p}\theta_{j}+\lambda_{\text{jp}}+\xi_{\text{jk}}.\; & \; \end{array}$$

Prior distributions should also be assigned to the additional parameters *γ*_*k*_ and *ξ*_*jk*_. These could be analogous to the priors for *α*_*p*_ and *λ*_*jp*_.

Notice that if we are interested in adjusting by continuous covariates we should create some categories before including them into the model. One possibility is to create some categories using tertiles or quartiles (e.g. when measuring the exposure to the compsumtion to any nutrient) or use a priori cut-points (e.g. age can be categorized depending on the risk groups). A special case when an adjustment for continuous covariates is required appears in genetic studies when the population structure has to be considered. In these cases, principal component analysis (PCA) is used to determine subpopulation the structure [22]. Then association analysis between genetic markers and the disease is performed using logistic regression adjusted for the two principal components instead of using a chi-square test. In this case, after performing PCA and using any clustering method, individuals are classified into subpopulations. These subpopulations can be included in the model as previouly mentioned.

#### Estimation of model parameters

The JAGS software (available at http://mcmc-jags.sourceforge.net/) was used to carry out MCMC posterior sampling using the R package rjags[23]. We ran the sampler for 40,000 iterations and considered estimates based on the last 30,000 runs, allowing a burn-in of 10,000 iterations. Two chains were run for each of the models. Convergence was assessed from trace plots. We also used the “potential scale reduction factor” diagnostic proposed by Gelman and Rubin [24].

MCMC is computationally intensive, even more in the case of analyzing genetic data where normally thousands of genes are analyzed. To overcome this difficulty we also used the Integrated Nested Laplace Approximation (INLA) approach to make statistical inference of our model. INLA provides a fast (it gives answers in minutes when MCMC requires hours and days) deterministic alternative to MCMC [25]. The only difference between both approaches is that the model based on INLA replaces the t distributions with Normals. This, in principle, could shrink CNV-disease risk associations more than the original model, but it runs much faster and it can be applied to GWAS. In any case, the t distribution can easily be incorporated when available for INLA (http://www.r-inla.org/). We have developed an R package called bayesGen that incorporates both estimating processess as well as some tools for displaying model parameters and evaluating model convergence. The package is available at http://www.creal.cat/jrgonzalez/software.htm.

## Results

### Genomic differences between human populations

Armengol et al. [16] showed some CNV loci that are present with different frequencies accross individuals belonging to three human populations (YRI-Yoruba in Ibadan, Nigeria, CEU-Utah residents with ancestry from Northern and Western Europe; and CHB/JPT-Han Chinese in Beijing, China and Japanese in Tokyo, Japan), representatives of sub-Saharan Africa, Europe and East Asia, respectively. The authors, in a preliminary step, used aCGH and BAC-based platforms to identify CNV loci with different frequencies in the three populations using pools of individuals. This yielded a total of 111 loci whose copy number state frequencies differed among populations. In order to confirm the changes detected with the aCGH platforms, they performed validation experiments using MLPA on individual DNAs from the HapMap samples. In total they analyzed 152 CNV loci (genes). Overall, they found 33 CNV loci that were specific to any of the three populations after applying standard statistical tests (*χ*^2^ or Fisher tests).

The final data set we use for illustration purposes consists of 120 CNV loci (we removed 32 CNV loci that were not variable among populations) and 261 individuals (56 CEU, 58 YRI and 147 CHB/JPT) belonging to the MLPA experiment. Therefore, our data consists of a 261 × 120-dimensional matrix with values corresponding to the observed copy number status *X*_*ijp *_∈ {0,1,2,3,4}. After aggregating the counts of each number of copies over the individuals in each population for each CNV loci we fit the model 2 to the aggregated data *Y*_*jp *_where *j *= 1,…,120 and *p*∈{CEU,*YRI*,*CHB*/*JPT*}. Using the bayesCNVassoc function in the bayesGenR package we ran two chains of 200,000 iterations. We discarded the first 20,000 and kept every 50 to reduce the autocorrelation in the chains. Inference is therefore based on (thinned) samples of size 4,000. We assessed convergence using graphical techniques and the Gelman-Rubin method and no symptoms of non-convergence were detected. To keep the false discovery rate under control when evaluating whether a specific component was statistically significant or not, we computed credible intervals at 99.98% level (in the frequentist framework this would be equivalent to a Bonferroni correction 0.05/120∼0.0002) for *λ*_*jp*_’s.

**Figure 2:**
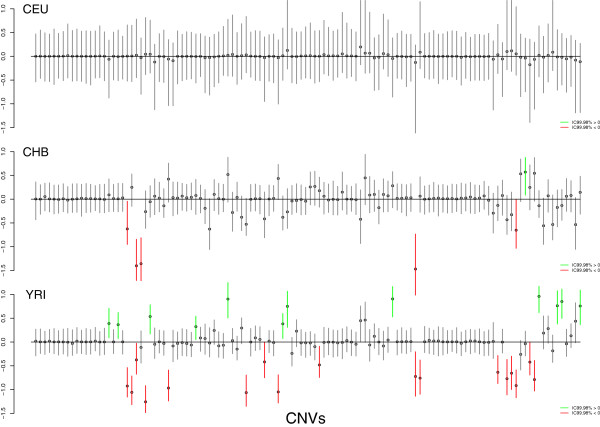
**Estimates of specific components, *λ*_*jp*_, for each CNV and each human populations belonging to HapMap data example.** Each point represents the posterior medians, while segments show its 99.98% credibility intervals. CNVs that are statistically significant specific of each population are coloured in red (gains) and blue (losses).

Table 1 shows the estimates for the population-specific intercepts *α*_*p*_ for the shared component model assuming a symmetric formulation. The specific intercept for all three populations, *α*_*p*_, is around 2 as expected. The shared component, *β*’s, are all 0. This is indicating that populations are sharing CNV loci frequencies. Regarding the specific component for each population we found that only 31 CNV loci were population-specific (Figure 2). By looking at the estimates of *ν*_*p*_ we observe that *ν*_*CEU*_ = 0.0756, while *ν*_CHB/*JPT*_ = 0.0306 and *ν*_YRI_ = 0.0362. This indicates that there is more variability among european individuals, which decreases the power of finding any specific CNV locus for european population. Trace plots and Gelman-Rubin scale reduction factor indicate good convergence of MCMC parameter estimates (see Additional file 1: Figures S1-S4 and Additional file 1: Table S1).

**Table 1:** Posterior median and 95% credibility intervals for population-specific intercepts corresponding to HapMap example

**Group**	**Parameter**	**median (95%CI)**
CEU	*α*_1_	1.95 (1.90, 2.02)
YRI	*α*_2_	1.99 (1.94, 2.04)
CHB/JPT	*α*_3_	1.97 (1.93, 2.03)

Armengol et al. [16] found 33 population-specific CNV loci after using *χ*^2^ or Fisher tests. In order to compare the performance of both approaches we tested the existence of population stratification (i.e. genetic differences among individuals) using a principal component analysis (PCA) as suggested in [22]. Armengol et al. estimated that 30% of the total variance is explained by the two first principal components (PC1 16.6%, and PC2 13.4%) using 33 CNV loci. In our case, with only 31 CNV loci, the two first principal components explain a 38.3% of the total variability (PC1 22.1%, and PC2 16.2%) indicating that our subset of variants discriminates better the individuals.

### Specific CNV loci associated with response to treatment in ovarian cancer

**Figure 3:**
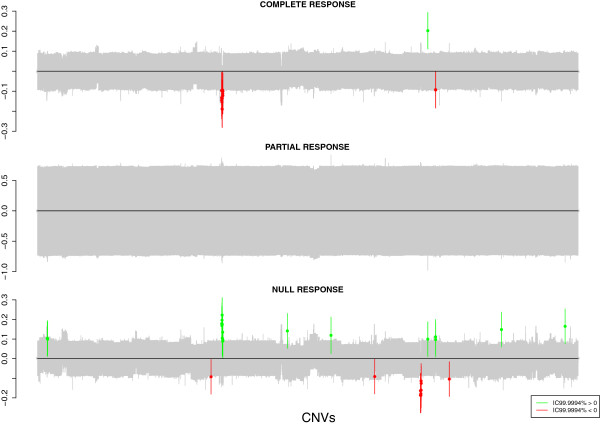
**Estimates of specific components, *λ***_***jp***_**, for each CNV and each group of individuals depending on response to treatments belonging to ovarian cancer example.** Each point represents the posterior medians, while segments show its 99.9994% credibility intervals. CNVs that are statistically significant specific of each population are coloured in green (gains) and red (losses).

This data set contains 8587 CNV loci and 456 individuals. The number of observed copies ranged from 0 to 6. This example was analyzed using INLA configuration of *bayesGen* package. As in the previous example, false discovery rate was controlled by computing credible intervals at a 99.9994% level (Bonferroni correction). Table 2 shows the estimates for the group-specific intercepts *α*_*p*_ for the shared component model under a symmetric formulation (e.g. no control group). Again, as expected, these intercepts are around 2. Regarding the specific components, we observe that only 57 CNV loci are statistically significant. As previouly mentioned, we were expecting a little number of CNV loci that are specific for each group, since analyzed individuals belong to the same ethnicity. HapMap data showed about 20% of CNV loci to be specific of each subgroup (33 out of 152 detected in [16]) while in this example only about 1% of CNV loci (57 out of 8587) are significantly associated with any of the three types of response to treatment. The complete list of specific CNV loci for each group can be found in Additional file 1: Table S2. Figure 3 shows *λ*_*jp*_ estimates. This figure illustrates those CNVs that are specific to get each response after treatment.

**Table 2:** Posterior median and 95% credibility intervals for population-specific intercepts corresponding to ovarian cancer example

**Group**	**Parameter**	**median (95%CI)**
Complete response	*α*_1_	2.00 (1.98, 2.03)
Partial response	*α*_2_	1.99 (1.97, 2.01)
Null response	*α*_3_	1.99 (1.97, 2.01)

### Simulation Studies

In real datasets we can only illustrate the methods, the truth about which CNV loci are really associated with each group is unknown. In order to evaluate our proposed method we carried out a small-scaled simulation study that mimics the real data analysis presented in previous section. We considered three different groups and 500 and 2,000 CNV loci. Only two of the CNVs were in a different proportion for one population (i.e. these two CNV loci were specific for such group of individuals). We simulated 3 different scenarios for the trully associated CNV loci. The first one considers that the two CNV loci are highly associated with one of the populations (OR=2.0), the second one considers a moderate increase on risk (OR=1.5), while the third one is designed to study the performance of our proposed method in a low risk scenario (OR=1.2). The simulation emulates a likely association between thousands of genes and disease. In genetic studies only a few of the analyzed genes are trully associated with the phenotype of interest. For instance, the WTCCC analyzed 3,432 CNV loci among different diseases and only found 3 loci associated with disease [21].

The copy number status for the loci were simulated considering two types of CNV data. The first one assumes that CNVs were common, meaning that they can be tagged by SNPs ( i.e. analysis of CNPs). In this scenario the copy number status can only be {0,1,2}. This kind of data has been obtained by several authors when analyzing CNVs [7,26,27]. This particular scenario could also be modelled assuming that a common CNV locus follows a Binomial distribution and, hence, the model proposed in [15] could also be used. The main advantage of using our formulation is that it can also be applied when CNV loci are not in HWE since the only assumption made is that the mean of the observed number of copies follows a gaussian distribution. This holds in general due to the central limit theorem as we are summing the number of copies for each group of individuals. The second scenario considers polymorphic CNV loci taking values {0,1,2,3,4,5,6}. This scenario tries to mimic situations in which complex CNVs are analyzed. In both cases we simulated CNV loci assuming Hardy-Weinberg equilibrium. The allelic frequencies were randomly selected from *U*(0.01, 0.1) trying to reflect the fact that most CNVs are rare CNVs. In addition, in order to assess the performance when analyzing CNPs as in [7], we also performed the same simulations assuming that allelic frequencies between 0.05 and 0.5. We compared the results obtained from our proposed Bayesian shared component model with those obtained with a *χ*^2^ test, a non-parametric Kruskall-Wallis test and a multinomial logistic regression comparing the null model versus the model including the CNV using the likelihood ratio test. Bonferroni correction was used in order to deal with multiple comparisons. We also computed corrected credible intervals for the specific components. Given that the Bonferroni-like correction requires estimation of extreme percentiles for the posterior distribution, which are difficult to be obtained from MCMC samples, we computed a credible interval based on the normal approximation. Finally, we considered the posterior probability as an alternative criterion to detect significant CNV loci. We compared the different approaches by computing the true positive and negative rates (TPR and TNR, respectively) in 500 simulations.

Table 3 shows the TPR and FPR for the different methods in the case of analyzing common CNVs with allelic frequencies between 0.01 and 0.1. Across all scenarios, as expected, the TPR decreases when the ORs for the significant CNV loci decrease. The TPR are almost 100% in all cases since only two of the CNV loci (500 or 2,000) were simulated with a signal different from 0. We observed that the Bayesian shared component model outperforms the other methods in the case of having low and moderate risk effects. This finding is important since common CNVs can be tagged by SNPs and their risks are expected to be about 1.15-1.45. For example, in the context of CNVs that can be tagged by SNPs, de Cid et al. [7] found that the risk of having one copy of the LCE gene increased by 41% the chance of having psoriasis. We finally noticed that non-parametric tests are not able to detect the two significant CNV loci in any situation, suggesting that such methods are not a good choice for the analysis of CNV data with a very small number of significant signals. On the other hand, Table 4 shows the TPR and FPR in the case of analyzing complex/polymorphic CNVs. Overall, the results are the same as those obtained for the case of analyzing common SNPs, showing even more differences between Bayesian model and the other methods. This can be explained by the fact that by simulating CNV loci with number of copies between 0 and 6, the number of individuals in each category is reduced. In this situation, the power of using methods based on the observed number of individuals in each category decreases. Additional file 1: Tables S3 and S4 show the results for the same simulations when allelic frequencies were simulated ranging from 0.05 and 0.5. The conclusions are the same and, as expected, the only difference is that the TPR and the TNR increase because allelic frequencies are higher.

**Table 3:** Results for the simulation study for the case of having common CNVs

					**Bayesian Shared Model**		
				**Multinomial**	**Posterior**	**Normal**	**Posterior**
	**# SNPs**	***χ***^**2**^	**K-W**	**regression**	**Distribution**	**Approximation**	**Probability**
high risk scenario (OR=2.0)							
TPR	2000	100.00	0	100.00	100.00	100.00	100.00
TNR	2000	100.00	100.00	100.00	99.98	99.99	99.96
TPR	500	100.00	0	100.00	100.00	100.00	100.00
TNR	500	99.73	100.00	99.73	99.99	99.95	99.80
moderate risk scenario (OR=1.5)							
TPR	2000	60.25	0	56.75	75.25	75.50	75.00
TNR	2000	99.95	100.00	99.95	99.98	99.99	99.95
TPR	500	69.25	0	67.50	96.25	96.25	95.75
TNR	500	99.81	100.00	99.81	99.96	99.99	99.98
low risk scenario (OR=1.2)							
TPR	2000	0.75	0	0.75	10.50	10.25	10.25
TNR	2000	99.99	100	99.9	100.00	100.00	99.98
TPR	500	1.50	0	3.25	25.25	26.50	25.50
TNR	500	99.99	100	99.99	99.99	99.99	99.98

Results for the simulation described in Simulation Studies Section for the case of having common CNVs with major allele frequency simulated from U(0.01, 0.1). The different scenarios are described in that section. We compare four different approaches: *χ*^2^ test, Kruskall-Wallis (K-W), Multinomial regression using likelihood ratio test, and our proposed Bayesian model. The comparison was based on computing the True Positive and Negative Rates, TPR and TNR respectively. Results are expressed in %.

**Table 4:** Results for the simulation study for the case of having polymorphic CNVs

					**Bayesian Shared Model**		
				**Multinomial**	**Posterior**	**Normal**	**Posterior**
	**# SNPs**	***χ***^**2**^	**K-W**	**regression**	**Distribution**	**Approximation**	**Probability**
moderate risk scenario (OR=2.0)							
TPR	2000	48.50	0	52.25	75.25	74.25	75.50
TNR	2000	100.00	100	100.00	100.00	100.00	100.00
TPR	500	46.25	0	42.50	64.50	64.75	64.25
TNR	500	100.00	100	100.00	100.00	100.00	100.00
moderate risk scenario (OR=1.5)							
TPR	2000	30.25	0	35.45	58.50	58.50	57.75
TNR	2000	100.00	100	100.00	99.98	99.99	99.97
TPR	500	20.50	0	23.25	44.25	44.25	44.50
TNR	500	99.99	100	99.99	99.96	99.96	99.94
low risk scenario (OR=1.2)							
TPR	2000	0.70	0	0.70	20.25	20.25	20.75
TNR	2000	99.98	100	99.99	99.97	99.99	99.98
TPR	500	0.50	0	0.50	16.25	16.25	15.75
TNR	500	99.99	100	99.99	99.99	99.99	99.98

Results for the simulation described in Simulation Studies Section for the case of having polymorphic CNVs with major allele frequency simulated from U(0.01, 0.1). The different scenarios are described in that section. We compare four different approaches: *χ*^2^ test, Kruskall-Wallis (K-W), Multinomial regression using likelihood ratio test, and our proposed Bayesian model. The comparison was based on computing the True Positive and Negative Rates, TPR and TNR respectively. Results are expressed in %.

Regarding to computation time, we compared the required time to fit a model with 2,000 CNV loci and 3,000 individuals (1,000 for each of the 3 populations) and chi-square approach took 7sec, Kruskal-Wallis 28sec, multinomial logistic regression 7min 40sec, Bayesian model using INLA 1min 39sec and Bayesian model using MCMC 1h 10m. All computations were done in a workstation Dual Intel Xeon X5482 3,2GHz 2x6 Mb, Quad-Core with 32Gb RAM.

## Conclusions

Here we considered the problem of determining copy number variants that are specific to different subgroups of individuals or different subphenotypes when thousand of markers are analyzed and only a few of them are truly associated with a given group. We have demonstrated the utility of our model by analyzing two real datasets. One focuses on describing how to find specific CNV loci for the three major ethnic groups, while the second example illustrates how to detect specific CNV loci related to the response to treatment in patients diagnosed with ovarian cancer. We have implemented a Bayesian shared component model to decompose the observed variability in the number of copies of each CNV loci into two components: shared and specific. Simulation results showed a better performance than other existing methods.

We established the CNV loci that are specific to each group by computing credible intervals of the posterior mean of the specific components and their posterior probabilities. In order to avoid false positive results, we adopted a Bonferroni-like correction. Therefore, credible intervals require estimation of extreme percentiles. This may lead to some difficulties when using MCMC samples. Thus, we also calculated credible intervals based on normal approximation. Simulation studies showed that this method slightly outperforms the method based on percentiles.

The model has been formulated using a hierarchical structure. Therefore, it is straightforward to add further levels of hierarchy if needed. For instance, CNVs can be in the same pathway or may have the same function. Thus, this information can be incorporated in the model in order to estimate better the effect of each CNV locus, as described in [28]. This new structure could be easily incorporated into our model by introducing a new hierarchy on top of the CNV loci. There are several ways this could be done. One could be as follows: imagine that a CNV *j* is involved in pathway *g*. Then, we could simply replace the prior distribution

$$\lambda_{\text{jp}}∼t_{4}\left(0,\sigma_{p}^{2}\right)$$

by

$$\lambda_{\text{jp}}∼t_{4}\left(\omega_{\text{gp}},\sigma_{p}^{2}\right)$$

and then assign hyperpriors to the parameters *ω*_*gp *_that would pick up the variation at the pathway level. With this formulation, large values of *ω*_*gp*_ would indicate an association between pathway *g* and population *p*.

Our model considers that the number of copies for each CNV locus is measured without uncertainty, as considered by some authors [13,14]. In principle this could be a limitation, but this is a problem related to the technology used to obtain information about CNVs and calling algorithms. Notice that some of the CNV studies obtain information about CNVs using SNP array data [13,14] that are not designed to detect such type of markers. Nonetheless, several authors have pointed out that this will not be a problem with the use of Next Generation Sequencing (NGS) methods [29-31]. This technology is already capable of detecting CNVs by taking advantage of read mapping and having a very low false positive rate [30]. In addition, as NGS continues to improve as well as computational methods of CNV calling, the uncertainty surronding CNV calls will fall rapidly, making our method to be valid.

We conclude that our proposed model is useful to discover specific genetic variants for different subgroups of individuals. This could help in determining differences in disease predisposition or response to pharmaceutical treatments. Estimating model parameters can be very time consuming, however we have developed an R package (bayesGen) that not only includes MCMC methods but also a fast estimation of the posterior distribution based on INLA that provides estimates for a whole chromosome in a few minutes.

## Competing interests

The authors declare that they have no competing interests.

## Authors’ contributions

JRG designed and coordinated the study. JA developed the statistical model. CA implemented the estimating algorithms. JRG wrote the bayesGen R package and carried out data analysis and simulations. All authors contributed to the interpretation and discussion of the results. JA and JRG drafted the manuscript. All authors read and approved the final manuscript.

## Supplementary Material

Additional file 1 Supplementary tables and figures. Click here for file
