# Supplementary material for: Bayesian model to detect phenotype-specific genes for copy number data
Source: BMC Bioinformatics. 2012 Jun 13;13:130. doi: 10.1186/1471-2105-13-130 (PMC3576305; doi:10.1186/1471-2105-13-130)
Supplement: Additional file 1 — Supplementary tables and figures. [file 1471-2105-13-130-S1.pdf]

Supplementary information

Bayesian model to detect phenotype-specific genes for copy number data

Juan R Gonzalez[1,2,4,*], Carlos Abellán[3], Juan J Abellán[3,4]

1 Center for Research in Environmental Epidemiology (CREAL), Barcelona, Spain

2 Hospital del Mar Research Institute (IMIM), Barcelona, Spain

3 Joint Research Unit on Genomics and Health, Centre for Public Health Research (CSISP) and Cavanilles Institute for Biodiversity and Evolutionary Biology, University of Valencia, Valencia, Spain

4 CIBER Epidemiology and Public Health (CIBERESP), Spain

* Corresponding author: Juan R Gonzalez (jrgonzalez@creal.cat)

Simulation Studies

					Bayesian Shared Model		
	# SNPs	χ^2	K-W	Multinomial regression	Posterior Distribution	Normal Approximation	Posterior Probability
high risk scenario (OR=2.0)							
TPR	2000	100.00	0	100.00	100.00	100.00	100.00
TNR	2000	100.00	100	100.00	99.97	99.99	99.94
TPR	500	100.00	0	100.00	100.00	100.00	100.00
TNR	500	99.99	100	99.99	99.88	99.94	99.76
moderate risk scenario (OR=1.5)							
TPR	2000	55.25	0	55.50	71.50	71.50	77.00
TNR	2000	100.00	100	100.00	99.98	99.99	99.95
TPR	500	68.50	0	66.25	78.25	86.75	84.50
TNR	500	99.98	100	99.99	99.91	99.94	99.82
low risk scenario (OR=1.2)							
TPR	2000	14.25	0	14.50	22.50	23.50	28.75
TNR	2000	100.00	100	100.00	99.99	100.00	99.98
TPR	500	25.25	0	25.75	32.50	33.25	36.50
TNR	500	99.99	100	99.99	99.99	99.99	99.98

Supplementary Table S1: Results for the simulation described in the Simulation Results Section (main manuscript) for the case of having common CNVs with major allele frequency simulated from U(0.05, 0.5). The scenarios are described in that section. We compare four different approaches: χ^2 test, Kruskall-Wallis (K-W), Multinomial regression using likelihood ratio test, and our proposed Bayesian model. The comparison was based on computing the True Positive and Negative Rates, TPR and TNR respectively. Results are expressed in %

	# SNPs	χ^2	K-W	Multinomial regression	Bayesian Shared Model: Posterior Distribution	Bayesian Shared Model: Normal Approximation	Bayesian Shared Model: Posterior Probability
moderate risk scenario (OR=2.0)							
TPR	2000	58.25	0	62.25	85.25	84.25	86.25
TNR	2000	100.00	100	100.00	100.00	100.00	100.00
TPR	500	51.25	0	53.50	75.75	76.75	72.75
TNR	500	100.00	100	100.00	100.00	100.00	100.00
moderate risk scenario (OR=1.5)							
TPR	2000	40.0	0	50.50	69.50	70.25	70.50
TNR	2000	100.00	100	100.00	99.99	99.98	99.97
TPR	500	24.25	0	32.25	57.50	58.25	58.00
TNR	500	99.99	100	99.99	99.95	99.96	99.90
low risk scenario (OR=1.2)							
TPR	2000	9.25	0	11.75	22.75	23.75	24.00
TNR	2000	100.00	100	100.00	99.99	99.99	99.98
TPR	500	7.75	0	9.75	19.25	20.50	21.50
TNR	500	99.99	100	99.99	99.99	99.99	99.98

Supplementary Table S2: Results for the simulation described in the Simulation Studies Section (main manuscript) for the case of having polymorphic CNVs with major allele frequency simulated from U(0.05, 0.5) . The scenarios are described in that section. We compare four different approaches: χ^2 test, Kruskall-Wallis (K-W), Multinomial regression using likelihood ratio test, and our proposed Bayesian model. The comparison was based on computing the True Positive and Negative Rates, TPR and TNR respectively. Results are expressed in %

Table of specific CNVs for ovarian data analysis

Supplementary Table S3: Specific CNVs for each of the type of response for the ovarian cancer example. CNVs are annotated at the Database of Genomic Variants using the genome build GRCh37 (`http://projects.tcag.ca/variation/downloads/variation.hg19.v10.nov.2010.txt`). Last three columns despite the specific effect (lambda) and confidence intervals (inf, sup) at 99.9994% level

CNV	group	inf	lambda	sup
Variation_66653	COMPLETE RESPONSE	-0.19	-0.10	-0.01
Variation_66654	COMPLETE RESPONSE	-0.22	-0.13	-0.04
Variation_66655	COMPLETE RESPONSE	-0.24	-0.15	-0.06
Variation_66656	COMPLETE RESPONSE	-0.24	-0.14	-0.05
Variation_66657	COMPLETE RESPONSE	-0.24	-0.14	-0.05
Variation_66658	COMPLETE RESPONSE	-0.23	-0.14	-0.05
Variation_66659	COMPLETE RESPONSE	-0.23	-0.14	-0.05
Variation_66660	COMPLETE RESPONSE	-0.25	-0.15	-0.06
Variation_66661	COMPLETE RESPONSE	-0.28	-0.19	-0.10
Variation_66662	COMPLETE RESPONSE	-0.23	-0.14	-0.04
Variation_66664	COMPLETE RESPONSE	-0.28	-0.19	-0.10
Variation_66665	COMPLETE RESPONSE	-0.23	-0.14	-0.04
Variation_66669	COMPLETE RESPONSE	-0.18	-0.09	-0.00
Variation_66671	COMPLETE RESPONSE	-0.21	-0.12	-0.03
Variation_66672	COMPLETE RESPONSE	-0.20	-0.11	-0.02
Variation_66673	COMPLETE RESPONSE	-0.20	-0.11	-0.02
Variation_66677	COMPLETE RESPONSE	-0.21	-0.12	-0.03
Variation_66678	COMPLETE RESPONSE	-0.19	-0.10	-0.01
Variation_70983	COMPLETE RESPONSE	0.11	0.20	0.29
Variation_71152	COMPLETE RESPONSE	-0.18	-0.09	-0.00
Variation_71154	COMPLETE RESPONSE	-0.18	-0.09	-0.00
Variation_63371	NULL RESPONSE	0.01	0.10	0.19
Variation_63376	NULL RESPONSE	0.01	0.10	0.19
Variation_63378	NULL RESPONSE	0.01	0.10	0.19
Variation_66452	NULL RESPONSE	-0.18	-0.09	-0.00
Variation_66656	NULL RESPONSE	0.09	0.18	0.27
Variation_66657	NULL RESPONSE	0.09	0.18	0.27
Variation_66658	NULL RESPONSE	0.08	0.17	0.26
Variation_66659	NULL RESPONSE	0.08	0.17	0.26
Variation_66660	NULL RESPONSE	0.11	0.20	0.29
Variation_66661	NULL RESPONSE	0.09	0.18	0.27
Variation_66662	NULL RESPONSE	0.13	0.22	0.31
Variation_66664	NULL RESPONSE	0.09	0.17	0.26
Variation_66665	NULL RESPONSE	0.02	0.10	0.19
Variation_66672	NULL RESPONSE	0.05	0.14	0.22
Variation_66673	NULL RESPONSE	0.01	0.10	0.18
Variation_66677	NULL RESPONSE	0.00	0.09	0.18
Variation_67889	NULL RESPONSE	0.05	0.14	0.23
Variation_68999	NULL RESPONSE	0.03	0.12	0.21

Variation_69898	NULL RESPONSE	-0.18	-0.09	-0.00
Variation_70823	NULL RESPONSE	-0.28	-0.19	-0.10
Variation_70824	NULL RESPONSE	-0.25	-0.16	-0.07
Variation_70825	NULL RESPONSE	-0.25	-0.16	-0.07
Variation_70826	NULL RESPONSE	-0.28	-0.19	-0.10
Variation_70829	NULL RESPONSE	-0.28	-0.19	-0.10
Variation_70830	NULL RESPONSE	-0.28	-0.19	-0.10
Variation_70831	NULL RESPONSE	-0.28	-0.19	-0.10
Variation_70832	NULL RESPONSE	-0.27	-0.18	-0.09
Variation_70838	NULL RESPONSE	-0.20	-0.11	-0.03
Variation_70839	NULL RESPONSE	-0.25	-0.16	-0.07
Variation_70841	NULL RESPONSE	-0.21	-0.13	-0.04
Variation_70983	NULL RESPONSE	0.01	0.10	0.19
Variation_71152	NULL RESPONSE	0.02	0.11	0.20
Variation_71154	NULL RESPONSE	0.01	0.10	0.19
Variation_71455	NULL RESPONSE	-0.19	-0.10	-0.01
Variation_72518	NULL RESPONSE	0.06	0.15	0.24
Variation_73808	NULL RESPONSE	0.08	0.17	0.25

Checking MCMC convergence for HapMap data analysis

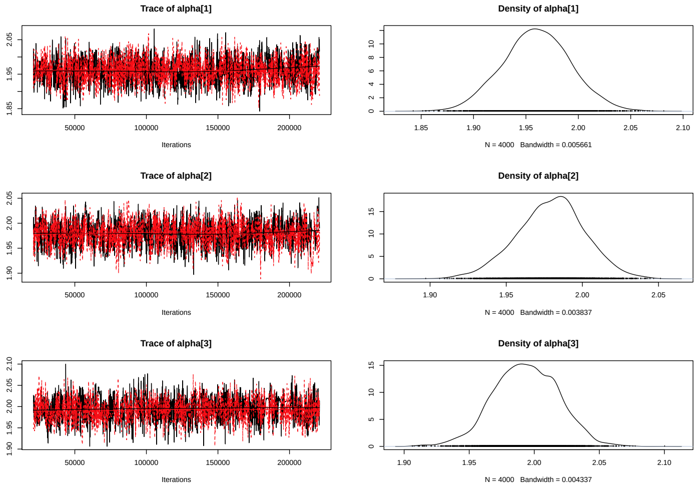

Supplementary Figure S1: Trace plot for α parameter estimates. Red and black lines represents each of the two MCMC chains

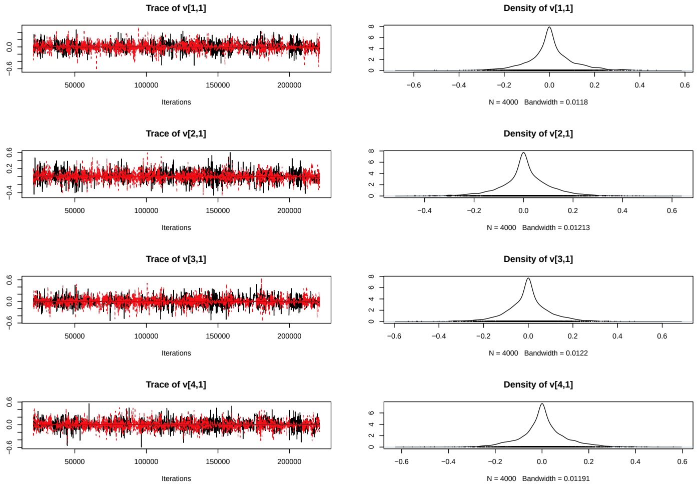

Supplementary Figure S2: Trace plot for some selected λ parameter estimates. Red and black lines represents each of the two MCMC chains

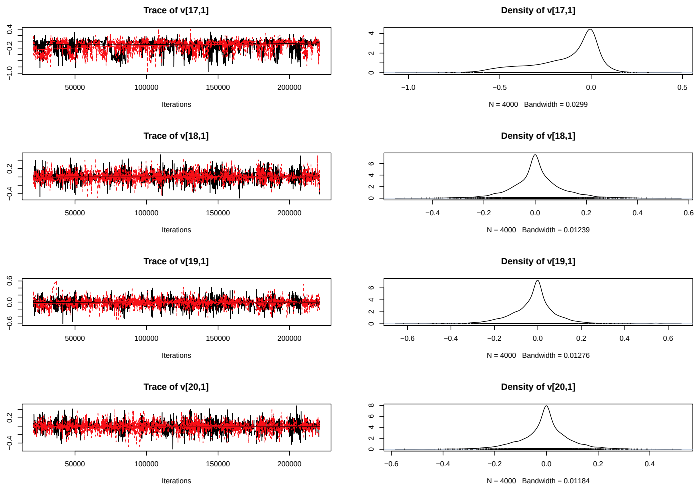

Supplementary Figure S3: Trace plot for some selected λ parameter estimates. Red and black lines represents each of the two MCMC chains

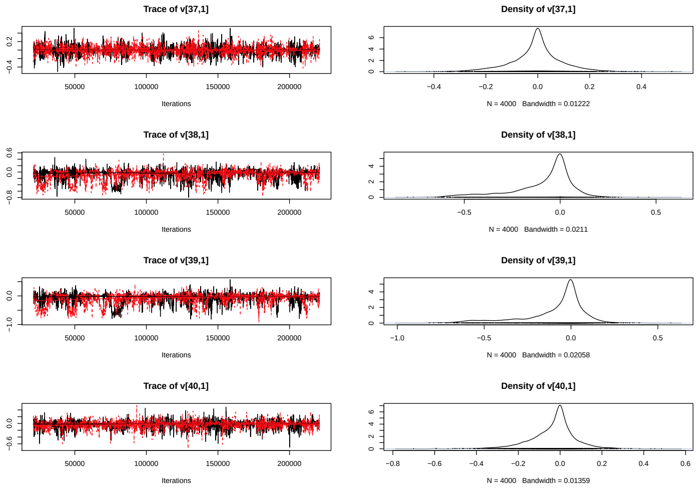

Supplementary Figure S4: Trace plot for some selected λ parameter estimates. Red and black lines represents each of the two MCMC chains

Supplementary Table S4: Gelman-Rubin scale reduction factor estimates for α and λ parameter estimates. Values around 1 indicates good convergence

	Point est.	Upper C.I.
alpha[1]	1.01	1.03
alpha[2]	1.00	1.01
alpha[3]	1.01	1.03
lambda[1,1]	1.00	1.00
lambda[2,1]	1.01	1.02
lambda[3,1]	1.00	1.01
lambda[4,1]	1.00	1.01
lambda[5,1]	1.01	1.02
lambda[6,1]	1.00	1.01
lambda[7,1]	1.01	1.01
lambda[8,1]	1.01	1.01
lambda[9,1]	1.00	1.00
lambda[10,1]	1.01	1.01
lambda[11,1]	1.00	1.00
lambda[12,1]	1.00	1.00
lambda[13,1]	1.02	1.06
lambda[14,1]	1.00	1.00
lambda[15,1]	1.00	1.00
lambda[16,1]	1.00	1.01
lambda[17,1]	1.02	1.05
lambda[18,1]	1.01	1.02
lambda[19,1]	1.01	1.03
lambda[20,1]	1.00	1.00
lambda[21,1]	1.02	1.05
lambda[22,1]	1.00	1.01
lambda[23,1]	1.02	1.04
lambda[24,1]	1.00	1.00
lambda[25,1]	1.00	1.00
lambda[26,1]	1.00	1.00
lambda[27,1]	1.00	1.02
lambda[28,1]	1.00	1.00
lambda[29,1]	1.00	1.02
lambda[30,1]	1.02	1.06
lambda[31,1]	1.01	1.02
lambda[32,1]	1.01	1.02
lambda[33,1]	1.00	1.00
lambda[34,1]	1.01	1.01
lambda[35,1]	1.00	1.01
lambda[36,1]	1.01	1.06
lambda[37,1]	1.00	1.00
lambda[38,1]	1.00	1.01
lambda[39,1]	1.00	1.00
lambda[40,1]	1.01	1.02
lambda[41,1]	1.00	1.01

lambda[42,1]	1.00	1.00
lambda[43,1]	1.00	1.00
lambda[44,1]	1.02	1.07
lambda[45,1]	1.01	1.01
lambda[46,1]	1.01	1.03
lambda[47,1]	1.01	1.05
lambda[48,1]	1.01	1.02
lambda[49,1]	1.00	1.01
lambda[50,1]	1.01	1.01
lambda[51,1]	1.00	1.00
lambda[52,1]	1.01	1.01
lambda[53,1]	1.00	1.00
lambda[54,1]	1.00	1.01
lambda[55,1]	1.00	1.00
lambda[56,1]	1.00	1.02
lambda[57,1]	1.00	1.01
lambda[58,1]	1.01	1.01
lambda[59,1]	1.00	1.00
lambda[60,1]	1.00	1.00
lambda[61,1]	1.00	1.00
lambda[62,1]	1.01	1.03
lambda[63,1]	1.00	1.00
lambda[64,1]	1.00	1.01
lambda[65,1]	1.00	1.01
lambda[66,1]	1.01	1.01
lambda[67,1]	1.01	1.01
lambda[68,1]	1.01	1.01
lambda[69,1]	1.00	1.00
lambda[70,1]	1.00	1.00
lambda[71,1]	1.00	1.00
lambda[72,1]	1.01	1.03
lambda[73,1]	1.01	1.04
lambda[74,1]	1.01	1.02
lambda[75,1]	1.01	1.03
lambda[76,1]	1.00	1.00
lambda[77,1]	1.00	1.01
lambda[78,1]	1.00	1.00
lambda[79,1]	1.00	1.00
lambda[80,1]	1.00	1.01
lambda[81,1]	1.00	1.00
lambda[82,1]	1.01	1.01
lambda[83,1]	1.00	1.00
lambda[84,1]	1.01	1.06
lambda[85,1]	1.01	1.03
lambda[86,1]	1.00	1.00
lambda[87,1]	1.00	1.00
lambda[88,1]	1.00	1.01

lambda[89,1]	1.00	1.00
lambda[90,1]	1.00	1.02
lambda[91,1]	1.00	1.00
lambda[92,1]	1.00	1.00
lambda[93,1]	1.00	1.00
lambda[94,1]	1.00	1.01
lambda[95,1]	1.01	1.03
lambda[96,1]	1.00	1.01
lambda[97,1]	1.00	1.00
lambda[98,1]	1.00	1.01
lambda[99,1]	1.01	1.01
lambda[100,1]	1.02	1.03
lambda[101,1]	1.00	1.00
lambda[102,1]	1.00	1.00
lambda[103,1]	1.00	1.00
lambda[104,1]	1.00	1.01
lambda[105,1]	1.01	1.03
lambda[106,1]	1.02	1.04
lambda[107,1]	1.02	1.05
lambda[108,1]	1.01	1.02
lambda[109,1]	1.01	1.02
lambda[110,1]	1.01	1.02
lambda[111,1]	1.01	1.04
lambda[112,1]	1.00	1.01
lambda[113,1]	1.00	1.00
lambda[114,1]	1.00	1.01
lambda[115,1]	1.00	1.01
lambda[116,1]	1.00	1.00
lambda[117,1]	1.01	1.05
lambda[118,1]	1.01	1.03
lambda[119,1]	1.01	1.01
lambda[120,1]	1.00	1.02
lambda[1,2]	1.00	1.00
lambda[2,2]	1.01	1.01
lambda[3,2]	1.00	1.00
lambda[4,2]	1.00	1.00
lambda[5,2]	1.00	1.01
lambda[6,2]	1.00	1.00
lambda[7,2]	1.00	1.00
lambda[8,2]	1.00	1.00
lambda[9,2]	1.00	1.00
lambda[10,2]	1.00	1.00
lambda[11,2]	1.01	1.01
lambda[12,2]	1.00	1.00
lambda[13,2]	1.01	1.03
lambda[14,2]	1.00	1.00
lambda[15,2]	1.00	1.00

lambda[16,2]	1.00	1.01
lambda[17,2]	1.01	1.05
lambda[18,2]	1.00	1.01
lambda[19,2]	1.00	1.01
lambda[20,2]	1.00	1.00
lambda[21,2]	1.02	1.03
lambda[22,2]	1.00	1.00
lambda[23,2]	1.01	1.04
lambda[24,2]	1.00	1.00
lambda[25,2]	1.00	1.01
lambda[26,2]	1.00	1.00
lambda[27,2]	1.00	1.02
lambda[28,2]	1.00	1.00
lambda[29,2]	1.00	1.01
lambda[30,2]	1.01	1.06
lambda[31,2]	1.01	1.05
lambda[32,2]	1.01	1.02
lambda[33,2]	1.00	1.00
lambda[34,2]	1.00	1.00
lambda[35,2]	1.00	1.01
lambda[36,2]	1.01	1.04
lambda[37,2]	1.00	1.00
lambda[38,2]	1.00	1.00
lambda[39,2]	1.01	1.01
lambda[40,2]	1.00	1.01
lambda[41,2]	1.00	1.00
lambda[42,2]	1.00	1.00
lambda[43,2]	1.02	1.02
lambda[44,2]	1.01	1.07
lambda[45,2]	1.00	1.00
lambda[46,2]	1.01	1.04
lambda[47,2]	1.01	1.04
lambda[48,2]	1.01	1.03
lambda[49,2]	1.00	1.00
lambda[50,2]	1.00	1.00
lambda[51,2]	1.00	1.00
lambda[52,2]	1.01	1.01
lambda[53,2]	1.00	1.00
lambda[54,2]	1.00	1.01
lambda[55,2]	1.00	1.00
lambda[56,2]	1.01	1.03
lambda[57,2]	1.00	1.00
lambda[58,2]	1.01	1.01
lambda[59,2]	1.00	1.00
lambda[60,2]	1.00	1.00
lambda[61,2]	1.01	1.02
lambda[62,2]	1.01	1.02

lambda[63,2]	1.00	1.00
lambda[64,2]	1.00	1.01
lambda[65,2]	1.00	1.01
lambda[66,2]	1.00	1.00
lambda[67,2]	1.00	1.00
lambda[68,2]	1.00	1.00
lambda[69,2]	1.00	1.00
lambda[70,2]	1.00	1.01
lambda[71,2]	1.00	1.00
lambda[72,2]	1.01	1.04
lambda[73,2]	1.00	1.01
lambda[74,2]	1.00	1.01
lambda[75,2]	1.00	1.01
lambda[76,2]	1.00	1.00
lambda[77,2]	1.00	1.00
lambda[78,2]	1.00	1.00
lambda[79,2]	1.00	1.00
lambda[80,2]	1.00	1.00
lambda[81,2]	1.00	1.00
lambda[82,2]	1.00	1.00
lambda[83,2]	1.00	1.00
lambda[84,2]	1.01	1.06
lambda[85,2]	1.00	1.01
lambda[86,2]	1.00	1.00
lambda[87,2]	1.00	1.00
lambda[88,2]	1.00	1.00
lambda[89,2]	1.00	1.00
lambda[90,2]	1.00	1.01
lambda[91,2]	1.00	1.01
lambda[92,2]	1.00	1.00
lambda[93,2]	1.00	1.00
lambda[94,2]	1.00	1.01
lambda[95,2]	1.01	1.02
lambda[96,2]	1.00	1.00
lambda[97,2]	1.00	1.00
lambda[98,2]	1.00	1.01
lambda[99,2]	1.00	1.00
lambda[100,2]	1.00	1.00
lambda[101,2]	1.00	1.00
lambda[102,2]	1.00	1.00
lambda[103,2]	1.00	1.00
lambda[104,2]	1.00	1.01
lambda[105,2]	1.01	1.03
lambda[106,2]	1.02	1.05
lambda[107,2]	1.02	1.07
lambda[108,2]	1.01	1.02
lambda[109,2]	1.01	1.05

lambda[110,2]	1.02	1.03
lambda[111,2]	1.00	1.02
lambda[112,2]	1.01	1.01
lambda[113,2]	1.00	1.00
lambda[114,2]	1.00	1.01
lambda[115,2]	1.00	1.00
lambda[116,2]	1.00	1.01
lambda[117,2]	1.01	1.03
lambda[118,2]	1.00	1.01
lambda[119,2]	1.00	1.00
lambda[120,2]	1.01	1.04
lambda[1,3]	1.01	1.03
lambda[2,3]	1.01	1.02
lambda[3,3]	1.00	1.01
lambda[4,3]	1.01	1.03
lambda[5,3]	1.00	1.01
lambda[6,3]	1.00	1.00
lambda[7,3]	1.00	1.01
lambda[8,3]	1.00	1.01
lambda[9,3]	1.00	1.01
lambda[10,3]	1.01	1.01
lambda[11,3]	1.01	1.03
lambda[12,3]	1.01	1.02
lambda[13,3]	1.00	1.01
lambda[14,3]	1.01	1.05
lambda[15,3]	1.01	1.02
lambda[16,3]	1.00	1.00
lambda[17,3]	1.00	1.01
lambda[18,3]	1.00	1.01
lambda[19,3]	1.00	1.01
lambda[20,3]	1.00	1.00
lambda[21,3]	1.01	1.01
lambda[22,3]	1.00	1.00
lambda[23,3]	1.01	1.01
lambda[24,3]	1.00	1.01
lambda[25,3]	1.00	1.00
lambda[26,3]	1.00	1.01
lambda[27,3]	1.00	1.00
lambda[28,3]	1.01	1.01
lambda[29,3]	1.00	1.00
lambda[30,3]	1.00	1.00
lambda[31,3]	1.00	1.00
lambda[32,3]	1.01	1.02
lambda[33,3]	1.00	1.00
lambda[34,3]	1.00	1.01
lambda[35,3]	1.00	1.01
lambda[36,3]	1.01	1.04

lambda[37,3]	1.01	1.02
lambda[38,3]	1.00	1.00
lambda[39,3]	1.00	1.00
lambda[40,3]	1.01	1.02
lambda[41,3]	1.00	1.01
lambda[42,3]	1.00	1.01
lambda[43,3]	1.00	1.02
lambda[44,3]	1.00	1.01
lambda[45,3]	1.01	1.01
lambda[46,3]	1.00	1.01
lambda[47,3]	1.00	1.00
lambda[48,3]	1.00	1.00
lambda[49,3]	1.00	1.01
lambda[50,3]	1.00	1.01
lambda[51,3]	1.00	1.00
lambda[52,3]	1.00	1.01
lambda[53,3]	1.01	1.02
lambda[54,3]	1.00	1.01
lambda[55,3]	1.00	1.01
lambda[56,3]	1.00	1.00
lambda[57,3]	1.00	1.00
lambda[58,3]	1.00	1.01
lambda[59,3]	1.00	1.01
lambda[60,3]	1.00	1.02
lambda[61,3]	1.01	1.03
lambda[62,3]	1.00	1.01
lambda[63,3]	1.00	1.00
lambda[64,3]	1.00	1.00
lambda[65,3]	1.00	1.01
lambda[66,3]	1.00	1.01
lambda[67,3]	1.01	1.01
lambda[68,3]	1.00	1.02
lambda[69,3]	1.00	1.01
lambda[70,3]	1.01	1.01
lambda[71,3]	1.00	1.00
lambda[72,3]	1.00	1.00
lambda[73,3]	1.00	1.01
lambda[74,3]	1.00	1.01
lambda[75,3]	1.00	1.01
lambda[76,3]	1.00	1.00
lambda[77,3]	1.00	1.01
lambda[78,3]	1.00	1.01
lambda[79,3]	1.00	1.00
lambda[80,3]	1.01	1.05
lambda[81,3]	1.00	1.01
lambda[82,3]	1.00	1.00
lambda[83,3]	1.00	1.00

lambda[84,3]	1.00	1.00
lambda[85,3]	1.00	1.01
lambda[86,3]	1.01	1.01
lambda[87,3]	1.00	1.00
lambda[88,3]	1.00	1.01
lambda[89,3]	1.00	1.00
lambda[90,3]	1.01	1.03
lambda[91,3]	1.00	1.01
lambda[92,3]	1.00	1.02
lambda[93,3]	1.00	1.02
lambda[94,3]	1.00	1.01
lambda[95,3]	1.01	1.02
lambda[96,3]	1.00	1.00
lambda[97,3]	1.00	1.01
lambda[98,3]	1.00	1.01
lambda[99,3]	1.01	1.02
lambda[100,3]	1.01	1.01
lambda[101,3]	1.00	1.00
lambda[102,3]	1.01	1.01
lambda[103,3]	1.00	1.00
lambda[104,3]	1.00	1.00
lambda[105,3]	1.00	1.00
lambda[106,3]	1.00	1.00
lambda[107,3]	1.00	1.01
lambda[108,3]	1.00	1.00
lambda[109,3]	1.00	1.00
lambda[110,3]	1.00	1.00
lambda[111,3]	1.00	1.00
lambda[112,3]	1.00	1.00
lambda[113,3]	1.00	1.01
lambda[114,3]	1.00	1.01
lambda[115,3]	1.00	1.00
lambda[116,3]	1.00	1.00
lambda[117,3]	1.00	1.00
lambda[118,3]	1.00	1.01
lambda[119,3]	1.00	1.00
lambda[120,3]	1.00	1.00
